# Design, Synthesis and Biological Evaluation of Novel Peptide-Like Analogues as Selective COX-2 Inhibitors

**Published:** 2018

**Authors:** Mohammad Ali Ahmaditaba, Mohammad Hassan Houshdar Tehrani, Afshin Zarghi, Sorayya Shahosseini, Bahram Daraei

**Affiliations:** a *Department of Pharmaceutical Chemistry, School of Pharmacy, Shahid Beheshti University of Medical Sciences, Tehran, Iran. *; b *Department of Toxicology, Faculty of Medical Sciences, Tarbiat Modares University, Tehran, Iran.*

**Keywords:** Peptide analogue, Solid phase peptide synthesis, Wang resin, COX-2 enzyme, Inhibitor

## Abstract

A new series of peptide-like derivatives containing different aromatic amino acids and possessing pharmacophores of COX-2 inhibitors as SO_2_Me or N_3 _attached to the *para* position of an end phenyl ring was synthesized for evaluation as selective cyclooxygenase-2 (COX-2) inhibitors. The synthetic reactions were based on the solid phase peptide synthesis method using Wang resin. One of the analogues, *i.e.*, compound 2d, as the representative of these series was recognized as the most effective and the highest selective COX-2 inhibitor with IC_50_ value of 0.08 μM and COX-2 selectivity index of 351.2, among the other synthesized compounds. Molecular docking study was operated to determine possible binding models of compound 2d to COX-2 enzyme. The study showed that the *p*-azido-phenyl fragment of 2d occupied inside the secondary COX-2 binding site (Arg^513^, and His^90^). The structure-activity relationships acquired disclosed that compound 2d with 4-(azido phenyl) group as pharmacophore and histidine as amino acid gives the essential geometry to provide inhibition of the COX-2 enzyme with high selectivity. Compound 2d can be a good candidate for the development of new hits of COX-2 inhibitors.

## Introduction

NSAIDs are one of the most popular drugs for curing inflammation which act through inhibition of prostaglandins biosynthesis. Cyclooxygenase (COX) is a key enzyme in transformation of arachidonic acid to prostaglandins (1). So far, three isoforms of this enzyme have been identified as COX-1, COX-2, and COX-3. COX-1 is expressed in many organelles and tissues and plays a protective role in digestive system, kidney, and homeostasis. But COX-2 isoform is only expressed when pathogenic conditions become created and consequently inflammatory process starts by this enzyme (2). COX enzymes have a key role in the biosynthesis of prostanoids and therefore, these enzymes involve in pain and fever mechanism (3). Because of non-selectivity of traditional NSAIDs, they have some side effects such as gastrointestinal and kidney dysfunctions due to the suppression of COX-1 and consequently blockade of different prostaglandins syntheses. 

 Therefore, it was thought that the use of selective COX-2 inhibitors could decrease these side effects. For this reason, many classes of selective COX-2 inhibitors have been identified and introduced as safe and reliable agents for treatment of inflammation, pain, and fever with fewer side effects like gastric ulceration, bleeding, and renal dysfunction. Most of the selective COX-2 inhibitors are linked to a class of vicinal diaryl heterocyclic ring system containing a SO_2_CH_3_, SO_2_NH_2_ or N_3_ as a crucial group at *para* position of an aryl ring that plays an important role as the pharmacophoric moiety of COX-2 selective inhibtors, the examples are celecoxib and rofecoxib (4-8). It is noteworthy that some of COX-2 inhibitors such as rofecoxib and valdecoxib are reportedly known as cardiovascular toxins and so have been withdrawn from the market (9). Accordingly, many researchers are eager to introduce new scaffolds of COX-2 inhibitors with higher potency and fewer side effects. For this reason new peptide templates could be good prospects for the future of selective COX-2 inhibitors. Recent studies in the development of peptides as therapeutics and increasing interests in this field resulted in around 140 therapeutic peptides which are now being assessed in clinical trials (10). In one study a series of dipeptides was reported as COX-2 Inhibitors. The peptides were checked experimentally by utilizing surface plasmon resonance (SPR). The authors recognized a dipeptide which could be a potential lead for another class of COX-2 inhibitors (11). In another study a series of fluorobenzoylated di- and tripeptides were reported as COX-2 inhibitors which showed considerable potency and selectivity compared with celecoxib (12). Structure-activity relationship surveys of this class of COX-2 inhibitors have demonstrated that a substitution of azide moiety, or SO_2_Me at *para* position of one of the phenyl rings often makes high selectivity on COX-2 inhibitory action (13-15). Therefore, it is attractive to develop novel selective COX-2 inhibitors with peptide structure which show anti-inflammatory effects.

**Figure 1:**
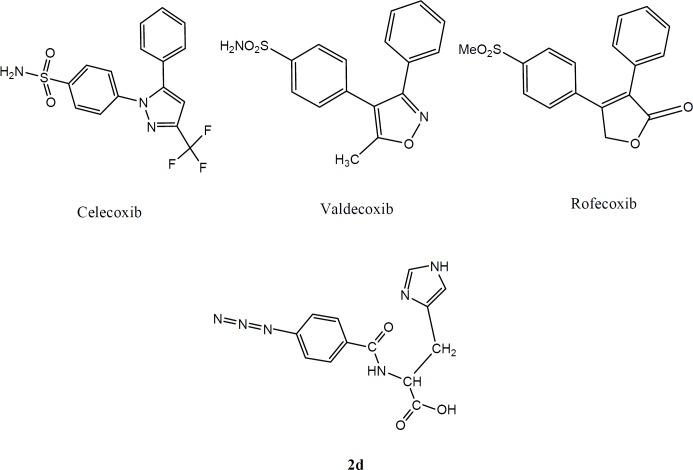
Some representative examples of selective cyclooxygenase-2 inhibitors and our designed molecule (2d

In the present work, we report the design and synthesis of a new dipeptide derivatives as COX-2 inhibitors containing SO_2_Me or N_3 _(azide) pharmacophores at *para* position of the phenyl ring (Figure 1). 

The design strategy was to combine pharmacophoric parts of COX-2 inhibitors with aromatic or cyclic amino acids to imitate the structure of COX-2 inhibitors.

## Experimental

*General*

N^α^ -Fmoc-protected amino acids, Wang resin, HOBt, DIC, piperazine, and trifluoroacetic acid were purchased from (Bachem and Sigma Aldrich). Peptide synthesis solvents, reagents, were analytical grade and provided from commercial sources (Merck, Germany) and used without further purification unless otherwise noted. Infrared spectra were acquired on a Perkin-Elmer 1420 ratio recording spectrometer. The mass spectral measurements were performed on a 6410 Agilent LCMS triple quadrupole mass spectrometer (LCMS) with an electrospray ionization (ESI) interface.

**Table 1:** *In-vitro* COX-1 and COX-2 enzyme inhibition data for compounds 1c-4d

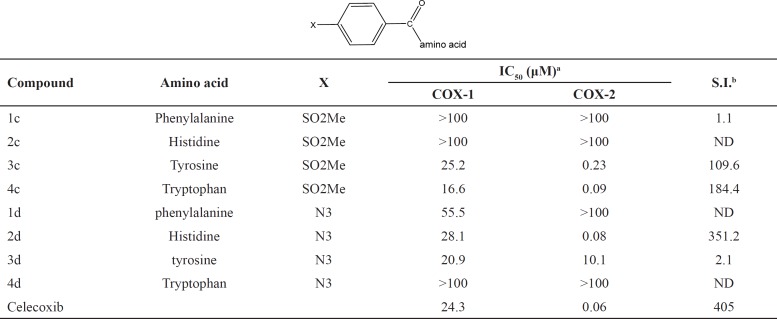

a Values are mean values of two determinations acquired using an ovine COX-1/COX-2 assay kit, where the deviation from the mean is < 10% of the mean value.

b *In-vit*ro COX-2 selectivity index (COX-1 IC_50_/COX-2 IC_50_).

**Figure 2:**
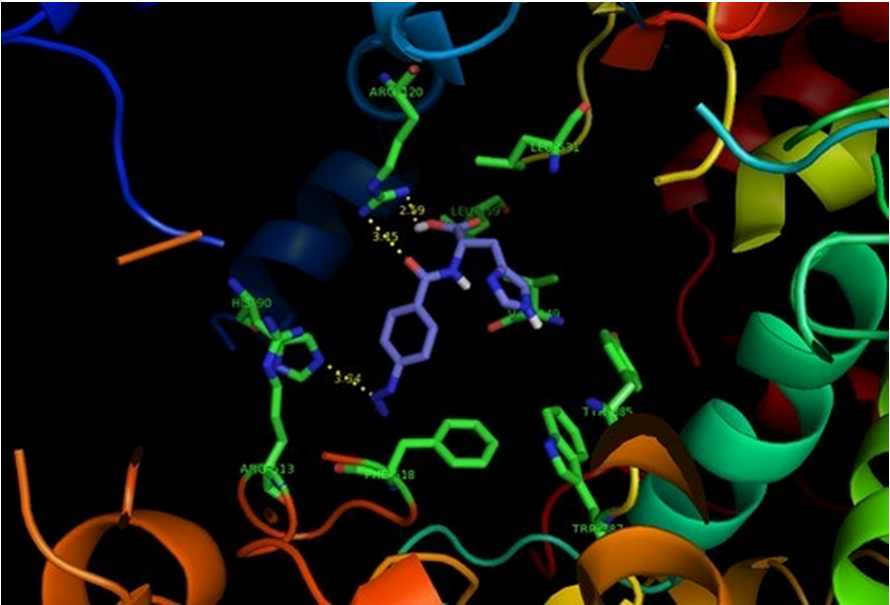
*p*-N_3_Bz -His (2d) (purple) docked in the active site of 6COX . Hydrogen atoms of the amino acid residues have been removed to improve clarity

*Chemistry*

*General procedure for attaching the first amino acid*

The synthesis of dipeptides **(**1c-4d) was carried out according to the solid phase approach using standard Fmoc methodology in a manual reaction vessel. The first amino acid, N^α^ Fmoc-Xaa-OH, was linked onto the Wang resin (100–200 mesh, 1% DVB, 1 mmol/g) using HOBt 

(2 eq) and DIC (1 eq) as an activating agent and a catalytic amount of DMAP. The N^α^-Fmoc protecting group was removed by treating the protected peptide- resin with a 10% solution of piperazine in DMF (30 min) and the resin was then washed with DMF (5×).

*General procedure for preparation of peptide-like analogues of COX-2 inhibitors (1c-4d)*

The following reactant materials; 4-(methylsulfonyl) benzoic acid or 4-azidobenzoic acid (2.0 eq), DIC (1eq), HOBt (2 eq), were dissolved in DMF or DCM and added to the resin and shaked slowly. The coupling reaction time was 3 h. The peptide- resin was washed with DMF (3×), DCM (3×), and methanol (3×). The peptides were deprotected and cleaved from solid support with trifluoroacetic acid/DCM/anisole/triisopropyl-silane (50:45:2.5:2.5) for 2 h. The resin was filtered off and the crude peptide was precipitated by adding dropwise to cold diethyl ether. The residual ether was removed by evaporation and the product was lyophilized.

*General procedure for the preparation of 4-(methylsulfonyl) benzoic acid*

4-Methylthiobenzaldehyde (3 mL) was dissolved in THF (10 mL) and Oxone (10 g) in THF/water (30 mL, 50:50) was added. The mixture was stirred at room temperature for 24 h. After evaporation of THF, the residue was extracted with chloroform, washed with 10% aqueous sodium bicarbonate and dried with anhydrous sodium sulfate and then the solvent was evaporated. In many cases, off-white to pale yellow solid was formed. Yield: (70-94%).

*General procedure for preparation of 4-azido benzoic acid*

4-Aminobenzoic acid (1.64 g, 12 mmol) was dissolved in water (10 mL) and HCl (6 mL, 12N) was added. The mixture was stirred at room temperature for 1 h, then cooled down to 0-5 °C in an ice bath, and to which, an aqueous solution (10 mL) containing NaNO_2_ (0.89 g, 13 mmol) was added dropwise. After 5 min, a solution of sodium azide (0.0.84 g, 13 mmol) in water (10 mL) was added to the mixture and stirred for 5 min. The precipitate was filtered, and extracted with ethanol (20 mL), and dried by vaccum to give a light yellow solid (1.52 g, yield 75%). The crude solid was used for the next step of the synthesis.

*Molecular modeling*

Docking simulation was done to predict interaction of compounds (1c-4d) with COX-2 binding site (PDB code: 6COX). Docking studies were performed using AutoDock software version 4.2. The coordinates of the X-ray crystal structure of the selective COX-2 inhibitor SC-558 bound to the murine COX-2 enzyme was obtained from the RCSB Protein Data Bank (6COX) and hydrogens were added. All the ligand molecules were built using the Builder module and were energy minimized for 1000 interactions reaching a convergence of 0.01 kcal/molÅ. 

The energy minimized ligands were superimposed on SC-558 in the PDB file of 6COX, after which SC-558 was deleted. Searching for the favorable binding configuration between the small flexible ligands and the rigid protein was the purpose of docking study. For efficiency, protein residues with atoms greater than 6.0 Å from the docking box were removed. These docked structures were very similar to the minimized structures obtained initially. The quality of the docked structures was evaluated by measuring the intermolecular energy of the ligand-enzyme assembly (16).

*In-vitro cyclooxygenase (COX) inhibition assays*

The assay was performed using an enzyme chemiluminescent kit (Cayman chemical, MI, USA) according to the previously reported method (17). 

## Results and Discussion

*Chemistry*

Groups of peptide-like analogues of COX-2 inhibitors possessing a MeSO_2_ group or N_3_ at *para*-position of the phenyl ring containing different amino acids (phenylalanine, histidine, tryptophan, and tyrosine) were prepared to study the effect of these substituents on selectivity and potency of COX-2 inhibitory activity. 

*p-MeSO2Bz-Phe (1c): p-(methylsulfonyl)benzoyl-phenylalanine*

Yield: 72%; purity 85%; white solid; IR (KBr): ν (cm^-1^) 1324, 1154 (SO_2_), 1400-1600 (aromatic), 1740 (C=O); LC-MS (ESI) m/z: 447.1(M - 1). 

*p-MeSO2Bz -His (2c)*

Yield*: *78%*; *purity 79*%;* white solid; IR (KBr*): *ν (cm^-1^) 1305, 1141 (SO_2_), 1400-1600 (aromatic), 1704 (C=O); LC-MS (ESI) m/z: 335.7 (M- 1).

*p-MeSO*_2_ *Bz -Tyr (3c)*

Yield: 55%; purity 91%; white solid; IR (KBr): ν (cm^-1^) 1305, 1161 (SO_2_), 1400-1600 (aromatic), 1732 (C=O); LC-MS (ESI) m/z: 361.7 (M - 1).

*p-MeSO2Bz -Trp (4c)*

Yield: 57%; purity 70%; white solid; IR (KBr): ν (cm^-1^) 1305, 1144 (SO_2_), 1400-1600 (aromatic), 1727 (C=O); LC-MS (ESI) m/z: 384.6 (M-1).

*p-N3Bz-Phe (1d): p-azidobenzoyl-phenylalanine*

Yield: 65%; purity 81%; white solid; IR (KBr): ν (cm^-1^) 1400-1600 (aromatic), 1773 (C=O), 2148 (N_3_); LC-MS (ESI) m/z: 308.7 (M - 1).

*p-N3Bz -His (2d)*

Yield: 81%; purity 85%; semi yellow solid; IR (KBr): ν (cm^-1^) 1400-1600 (aromatic), 1758 (C=O), 2140 (N_3_); LC-MS (ESI) m/z: 298.7 (M - 1).

*p-N3Bz -Tyr (3d)*

Yield: 78%; purity 87%; white solid; IR (KBr): ν (cm^-1^) 1400-1600 (aromatic), 1725 (C=O), 2132 (N_3_); LC-MS (ESI) m/z: 325.1 (M - 1).

*p-N3Bz -Trp (4d)*

Yield: 61%; purity 65%; purple solid; IR (KBr): ν (cm^-1^) 1400-1600 (aromatic), 1727 (C=O), 2134 (N_3_); LC-MS (ESI) m/z: 347.7 (M - 1).

*Biological Studies*

SAR data (IC_50 _µM values) obtained by determination of the *in-vitro* ability of the synthesized compounds to inhibit the COX-1 and COX-2 isozymes showed that the position of the COX-2 SO_2_Me or N_3 _pharmacophore and the nature of the attaching amino acid were important on COX-2 inhibitory potency and selectivity. The outcome of COX-1/COX-2 inhibition assay is outlined in Table 1. SAR data (IC_50_ values) acquired by the calculation of the *in-vitro* potency (17) of the heading compounds to inhibit the COX-1 and COX-2 isozymes displayed that some of dipeptides (3c, 4c and 2d) were acceptable inhibitors of the COX-2 isozyme with IC_50_ values in the 0.08-0.23 μM range, and COX-2 selectivity indices in the 184.4-351.2 range. These results also indicated that the peptide derivatives containing *para* azido showed both better selectivity and potency for COX-2 inhibitory activity compared with SO_2_Me analogs. This may be explained by the hydrogen binding ability of nitrogen atoms in azide group for better interaction with the COX-2 active site. Therefore, the introduction of suitable substituents at *para* position of the phenyl ring combined with an aromatic amino acid moiety improved the selectivity and potency for COX-2 inhibitory activity. These data showed that the varied pharmacophores attached to *para* position of the phenyl ring and type of amino acid can influence both selectivity and potency for COX-2 inhibitory activity. Our results indicated that *p*-N_3_Bz -His (2d) showed the highest COX-2 selectivity Index (S.I. = 351.2) among the synthesized compounds which may be due to the better interaction with the COX-2 active site. The orientation and binding interactions of the most selective COX-2 inhibitor (2d) within the COX-2 binding site were predicted by a docking experiment. Docking the selective and potent COX-2 inhibitor (2d) into the COX-2 binding site (Figure 2) shows that the *para*-N_3_ substituent inserts into the secondary pocket present in COX-2 (Arg^513^, Phe^518^, Val^523^) and, furthermore, the acidic functional group of the docked compound shows a hydrogen bond with Arg^120^ (distance = 2.59 Å). In addition, docking shows hydrophobic pocket surrounding imidazole group by the side chains of residues Tyr^385^, Leu^359^ and Val^349^ which may explain the higher potency of compound (2d) compared with other analogs.

## Conclusions

A new class of dipeptides that are readily accessible via solid phase peptide synthesis was designed and synthesized for evaluation as COX-2 inhibitors. *In-vitro* enzyme inhibition structure-activity studies indicated that the azidophenyl moiety containing histidine structure is a suitable scaffold (template) to design COX-2 inhibitors.
